# Measurement of carbohydrates and organic acids in varieties of cheese using high‐performance liquid chromatography

**DOI:** 10.1002/fsn3.2438

**Published:** 2023-04-07

**Authors:** Mahmoud E. Ahmed, Ahmed R. A. Hammam, Abd El‐Fatah Ali, Khalid A. Alsaleem, Mohamed Salem Elfaruk, Dalia G. Kamel, Asmaa H. M. Moneeb

**Affiliations:** ^1^ Dairy Science Department Faculty of Agriculture Assiut University Assiut Egypt; ^2^ Dairy and Food Science Department South Dakota State University Brookings SD USA; ^3^ Department of Food Science and Technology Faculty of Agriculture Assiut University Assiut Egypt; ^4^ Department of Food Science and Human Nutrition College of Agriculture and Veterinary Medicine Qassim University Buraydah Saudi Arabia; ^5^ Medical Technology College Nalut University Nalut Libya

**Keywords:** carbohydrates, cheese, high‐performance liquid chromatography, lactose, organic acids

## Abstract

Lactose is converted to lactic acid through fermentation and ripening of cheese using starter cultures. The content of lactic acid and organic acids formed during storage of cheese is different based on the type of starter cultures, pH, processing, and storage conditions. The objective of this study was to determine the carbohydrates and organic acids of four different commercial cheese samples (Parmesan, Mozzarella, Swiss, and Cheddar cheese) using high‐performance liquid chromatography (HPLC). The lactose content in Cheddar cheese was significantly high (*p* < .05) as compared to Parmesan cheese while Mozzarella and Swiss cheese did not have lactose. However, galactose was low in Swiss cheese as compared to other cheese types, while glucose did not detect in all cheese samples. Organic acids such as citric, succinic, lactic, and butanoic acids were high in Parmesan cheese relative to other cheese types. Additionally, pyruvic and propanoic acids were high (*p* < .05) in Swiss cheese while acetic and orotic acids were elevated (*p* < .05) in Mozzarella cheese relative to other types of cheese.

## Graphical abstract

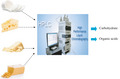

## 1 INTRODUCTION

Cheese is a dairy product obtained from milk coagulation using rennet and starter cultures. Cheese has many varieties that are different in flavors depending on the type of used milk and cultures. Once the milk is coagulated, the curd is separated by draining the whey. Then, the cheese is pressed and kept for ripening. Many changes are occurred during ripening to get the flavors. High‐Performance Liquid Chromatography (HPLC) is used to determine the components that resulted during ripening (Bouzas et al., 1991; Mullin & Emmons, 1997; Upreti et al., 2006; Zaky et al., 2017; Zeppa et al., 2001). The organic acids in cheeses can be measured precisely using HPLC. HPLC also can be utilized to monitor dairy products through fermentation or shelf life. The lactose is converted to lactic acid during fermentation, and then organic acids can result from the lactic acid. Organic acids are derived mainly from starter cultures and enzymes during ripening. Therefore, the objective of this study was to analyze and determine the organic acids of four different cheese samples, such as Parmesan, Mozzarella, Swiss, and Cheddar cheese.

## 2 MATERIAL AND METHODS

### 2.1 Sample preparation

Four varieties of cheese samples, including Parmesan, Mozzarella, Swiss, and Cheddar (1–3 months old) were obtained from the commercial market. Each cheese sample was prepared and analyzed in duplicates. The methodology was followed as described in several studies (Amamcharla & Metzger, 2011). Cheese samples were shredded and prepared for the HPLC (Beckman coulter, US) analysis. The buffer solution (0.01 N sulfuric acid) was tempered in the water bath at 70°C. Approximately 5 g of each cheese was weighed in a 50 ml centrifuge tube, and then 25 g of buffer solution was added in the tube. The samples were grinded using the homogenizer (model PT‐MR 2100, POLYTRON, Switzerland) at 12,000 rpm and then centrifuged (CR 412, JOUAN, UK) at 3,000 rpm at 5°C for 15 min. Once the centrifugation was done, the fat was solidified on the tube surface. The fat layer was removed and filtered into 125 ml Erlenmeyer flask using a filter paper (Whatman #1). A 0.5 ml of centrifugal filters (Millipore Corporation, Bedford, MA) was taking place into a 1.5 ml centrifuge tube and then centrifugated at 11,000 rpm for 10 min to remove any supernatant from the sample.

### 2.2 Mobile phase preparation

The mobile phase was filtered through a membrane using vacuum suction. The beaker was connected to a vacuum pump to reduce the pressure above the surface of the mobile phase and permit escape and subsequent removal of entrapped air from the mobile phase.

### 2.3 Priming the pumps

The drain valve was fully turned counterclockwise, and then the pump‐priming level turned down to prime lines position. The solvent was drawn slowly through the line by a syringe plunger with no air bubbles in the solvent line between the solvent bottle and the syringe. Then, the pump‐priming level turned up to prime pump position, and the syringe plunger was depressed through the liquid head and out the drain line to waste. When no bubbles are observed through the waste line, the priming level was turned to operate and the syringe plunger stopped depressing. The drain valve was not closed and this repeated for each solvent line. Then, the drain valve was closed by turning the valve knob fully clockwise.

### 2.4 Starting the instrument

**FIGURE 1:**
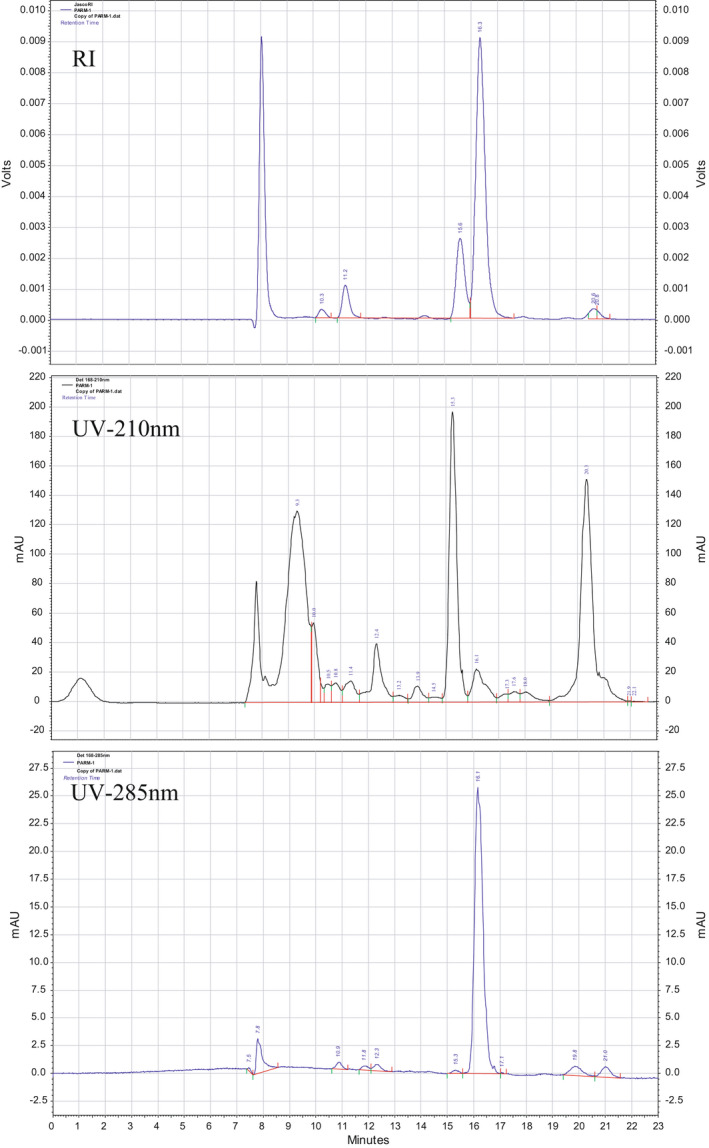
Chromatographs with area size and time retention obtained from JascoRI, UV‐210nm, and UV‐285nm

The instrument was turned on and then the flow rate was opened. First, the flow rate was set to 0.1 ml/min and clicked on to start the pump (Beckman Coulter, US) for 5 min. Then, the flow rate was set to 0.3 ml/min and clicked on to start the pump for 5 min. When the backpressure was stabled, the flow rate was switched to 0.6 ml/min. The PDA lamp was on and the RI detector turned on manually by pressing the front button. The system was left for 30 min to be stabilized. Afterward, the handle was switched from load to inject position and the sample was injected. The chromatographs from JascoRI, UV‐210nm, and UV‐285nm (Figure 1) were collected, and areas were calculated. The concentration of each compound was calculated. Each type of cheese was repeated in duplicates.

### 2.5 Statistical analyses

Results were analyzed by R software (R x64‐3.3.3, 9,205 NW 101st St, Miami, Florida, United States). All data were analyzed by ANOVA using a GLM for each variable. Mean separation was done using the least significant difference (LSD) comparison test when significant differences were detected at *p* < .05.

## 3 RESULTS AND DISCUSSION

**FIGURE 2:**
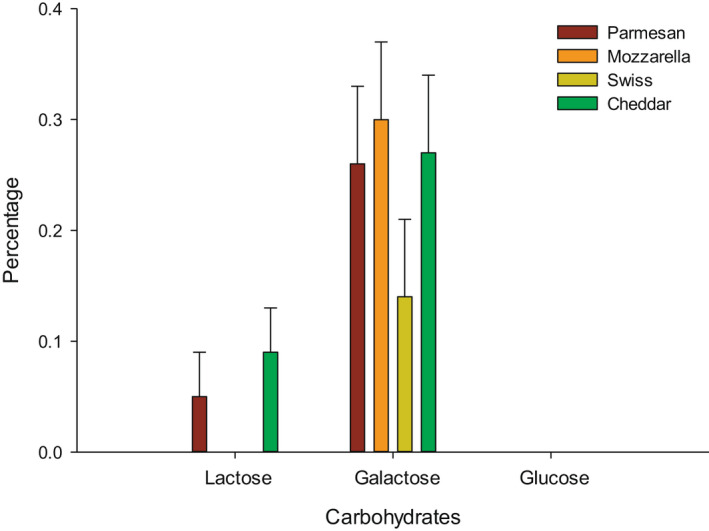
Percentage of carbohydrates in Parmesan, Mozzarella, Swiss, and Cheddar cheese determined using HPLC

**FIGURE 3:**
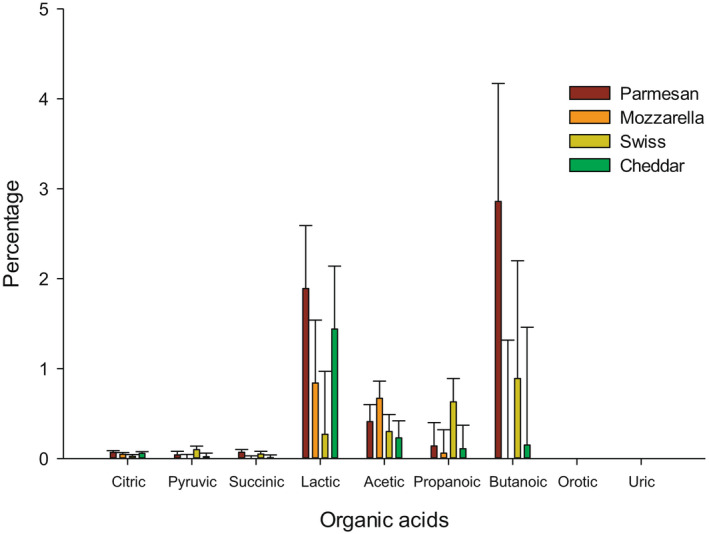
Percentage of organic acids in Parmesan, Mozzarella, Swiss, and Cheddar cheese determined using HPLC

The HPLC results for the four cheese samples are shown in Table 1. Also, Figure 2 is exemplified the percentage of carbohydrates resulted from cheese samples. Three different detectors were used to identify each concentration. RI detector was used to identify lactose, glucose, and galactose, which represent the carbohydrates in cheese samples. Figure 3 is presented the content of the organic acids in cheese samples obtained from HPLC. UV 210 was used to identify citric, pyruvic, succinic, lactic, acetic, propanoic, and butanoic acids, while orotic and uric acids were identified by UV 285, which present the organic acids.

**TABLE 1:** Percentage of carbohydrate and organic acids in Parmesan, Mozzarella, Swiss, and Cheddar cheese using HPLC

Sample	Parmesan	Mozzarella	Swiss	Cheddar	*SD*
Carbohydrate					
Lactose	0.05^b^	0.0^c^	0.0^c^	0.09^a^	0.04
Galactose	0.26^a^	0.30^a^	0.14^b^	0.27^a^	0.07
Glucose	0.0	0.0	0.0	0.0	0.0
Organic acid					
Citric	0.068^a^	0.047^ab^	0.023^b^	0.056^a^	0.02
Pyruvic	0.04^b^	0.005^d^	0.098^a^	0.02^c^	0.04
Succinic	0.07^a^	0.0^c^	0.05^ab^	0.01^bc^	0.03
Lactic	1.89^a^	0.84^c^	0.27^d^	1.44^b^	0.70
Acetic	0.41^b^	0.67^a^	0.30^c^	0.23^d^	0.19
Propanoic	0.14^b^	0.06^d^	0.63^a^	0.11^c^	0.26
Butanoic	2.86^a^	0.006^c^	0.89^b^	0.15^c^	1.31
Orotic	0.0001^c^	0.0066^a^	0.0016^b^	0.0020^b^	0.00
Uric	0.0	0.0	0.0	0.0	0.00

^a–d^Means in the same row not sharing a common superscript are different (*p* < .05).

Lactose was not found in Mozzarella and Swiss cheese, while it was 0.05 and 0.09% for Parmesan and Cheddar cheese, respectively (Figure 2). It has been reported that Cheddar cheese had 0.34%–0.35% of lactose after 2 weeks of ripening and this amount decreased to around 0.001% or did not present after 10 weeks of ripening (Upreti et al., 2006). The glucose content was not found in all cheese samples, and this was expected because glucose is utilized as a nutrient (easy to hydrolyze) for bacteria and it is the final result of lactose hydrolysis. Galactose was detected in Parmesan, Mozzarella, Swiss, and Cheddar cheese by 0.26, 0.30, 0.14, and 0.27%, respectively. The Galactose percentage in Cheddar cheese was similar to other studies that found after 5 weeks of ripening.

The citric acid was found in all cheese samples by 0.068, 0.047, 0.023, and 0.056% in Parmesan, Mozzarella, Swiss, and Cheddar cheese, respectively (Figure 3). The percentage of pyruvic acid was 0.04, 0.005, 0.098, and 0.02% for Parmesan, Mozzarella, Swiss, and Cheddar cheese, respectively. It has been found that Cheddar cheese could maintain 0.02% of pyruvic acid after 10 weeks of ripening (Upreti et al., 2006). Succinic acid was not detected in the Mozzarella cheese, but it was 0.07, 0.05, and 0.01% for Parmesan, Swiss, and Cheddar cheese, respectively. The lactic acid was higher in Parmesan and Cheddar cheeses as compared to Mozzarella and Swiss cheese. After 4–5 weeks of Cheddar cheese ripening, lactic acid was around 1.40% (Upreti et al., 2006), which refers to the age of the cheddar cheese in this study was around 4–5 weeks.

The Parmesan, Mozzarella, Swiss, and Cheddar cheese had 0.41, 0.67, 0.30, and 0.30%, respectively, for acetic acid, while the propanoic acid was 0.14, 0.06, 0.63, and 0.11%, respectively. Acetic acid did not expect to have 0.23%, which is higher than the results reported by Upreti during the ripening of cheddar cheese (<0.016%). Butanoic acid was higher in the Parmesan cheese than other cheeses. Cheddar cheese had 0.15% of butanoic acid which similar to Upreti results after 2 weeks of ripening. Parmesan cheese was expected to have 0.0001% orotic acid, while Mozzarella had the highest percentage of orotic acid 0.0066%. Lactic acid content was higher in the parmesan cheese than the other samples. Uric acid was expected to have a very low percentage (~0.0%) in all cheese samples, which was similar in cheddar cheese results (Upreti et al., 2006).

## 4 CONCLUSION

The objective of this study was to analyze and determine the carbohydrates and organic acids of four different cheese samples (Parmesan, Mozzarella, Swiss, and Cheddar cheese) using HPLC. We concluded that lactic acid and butanoic acids were higher in the Parmesan cheese as compared to other cheese samples. Mozzarella and Swiss cheese were free of lactose.

## AUTHOR CONTRIBUTIONS

**Mahmoud E. Ahmed:** Conceptualization (equal); Data curation (equal); Formal analysis (equal); Funding acquisition (equal); Investigation (equal); Methodology (equal); Project administration (equal); Resources (equal); Software (equal); Supervision (equal); Validation (equal); Visualization (equal); Writing‐original draft (equal); Writing‐review & editing (equal). **Ahmed R A** **Hammam:** Writing‐original draft (equal); Writing‐review & editing (equal). **Abd El‐Fatah Kotb Ali:** Formal analysis (equal); Investigation (equal); Visualization (equal); Writing‐original draft (equal). **Mohamed Salem** **Elfaruk:** Methodology (equal); Project administration (equal); Writing‐original draft (equal); Writing‐review & editing (equal). **Khalid** **Alsaleem:** Conceptualization (equal); Methodology (equal); Project administration (equal); Writing‐original draft (equal); Writing‐review & editing (equal). **Dalia G** **Kamel:** Methodology (equal); Project administration (equal); Supervision (equal); Writing‐original draft (equal); Writing‐review & editing (equal). **Asmaa H. M. Moneeb:** Conceptualization (equal); Data curation (equal); Formal analysis (equal); Funding acquisition (equal); Investigation (equal); Methodology (equal); Project administration (equal); Resources (equal); Software (equal); Supervision (equal); Validation (equal); Visualization (equal); Writing‐original draft (equal); Writing‐review & editing (equal).

## Data Availability

Research data are not shared.
